# Genome-wide identification of the *NHE* gene family in *Coilia nasus* and its response to salinity challenge and ammonia stress

**DOI:** 10.1186/s12864-022-08761-9

**Published:** 2022-07-20

**Authors:** Jun Gao, Zhijuan Nie, Gangchun Xu, Pao Xu

**Affiliations:** 1 Key Laboratory of Freshwater Fisheries and Germplasm Resources Utilization, Ministry of Agriculture, Freshwater Fisheries Research Center, Chinese Academy of Fishery Sciences, Wuxi, 214081 Jiangsu China; 2 Wuxi Fisheries College, Nanjing Agricultural University, Wuxi, 214081 Jiangsu China

**Keywords:** Na^+^-H^+^ exchangers, Chinese tapertail anchovy, Hypotonic stress, Hypertonic stress, High environmental ammonia (HEA), Gene expression

## Abstract

**Background:**

In aquatic environments, pH, salinity, and ammonia concentration are extremely important for aquatic animals. NHE is a two-way ion exchange carrier protein, which can transport Na^+^ into cells and exchange out H^+^, and also plays key roles in regulating intracellular pH, osmotic pressure, and ammonia concentration.

**Results:**

In the present study, ten *NHEs*, the entire *NHE* gene family, were identified from *Coilia nasus* genome and systemically analyzed via phylogenetic, structural, and synteny analysis. Different expression patterns of *C. nasus NHEs* in multiple tissues indicated that expression profiles of *NHE* genes displayed tissue-specific. Expression patterns of *C. nasus NHEs* were related to ammonia excretion during multiple embryonic development stages. To explore the potential functions on salinity challenge and ammonia stress, expression levels of ten *NHEs* were detected in *C. nasus* gills under hypotonic stress, hypertonic stress, and ammonia stress. Expression levels of all *NHEs* were upregulated during hypotonic stress, while they were downregulated during hypertonic stress. *NHE2* and *NHE3* displayed higher expression levels in *C. nasus* larvae and juvenile gills under ammonia stress.

**Conclusions:**

Our study revealed that *NHE* genes played distinct roles in embryonic development, salinity stress, and ammonia exposure. Syntenic analysis showed significant difference between stenohaline fish and euryhaline fishes. Our findings will provide insight into effects of *C. nasus NHE* gene family on ion transport and ammonia tolerance and be beneficial for healthy aquaculture of *C. nasus*.

**Supplementary Information:**

The online version contains supplementary material available at 10.1186/s12864-022-08761-9.

## Background

Na^+^/H^+^ exchanger (NHE) is a transmembrane protein that exists in all eukaryotic cells. Nine NHEs have been identified since human *NHE1* cDNA was successfully cloned [1]. Based on subcellular localization and phylogenic analysis, NHEs can be classified into plasmalemmal subgroup (NHE1–5, SLC9A1–5) and intracellular subgroup (NHE6–9, SLC9A6–9) in fish [2]. Plasmalemmal NHEs usually cooperate with bicarbonate transporter to regulate cytoplasmic pH, cell volume, and intracellular fluid secretion, thereby maintaining the balance of acid-base, electrolyte, and cell volume in the entire life system [3, 4]. Intracellular NHEs can not only transport Na^+^, Li^+^, and K^+^, but can also limit the excess acidification of organelles caused by vacuolar H^+^-ATPase (HA) [4, 5]. NHE1 has been demonstrated to be involved in cardiac remodeling and myocardial fibrosis [6]. NHEs, such as NHE6 and NHE9, can inhibit proliferation and migration in a variety of tumors [7].

In aquatic environments, pH, salinity, and ammonia concentration are extremely important for aquatic animals. NHE is a two-way ion exchange carrier protein, which can transport Na^+^ into cells and exchange out H^+^, and also plays key roles in regulating intracellular pH, osmotic pressure, and ammonia concentration [8–11]. In fish, the plasma pH cannot be regulated via excreting CO_2_. Thus, H^+^ and HCO_3_^−^ transport in fish gills plays a critical role in acid-base regulation [10, 12]. In hypertonic environments, fish excrete metabolic acids through the apical NHE, which is generally believed to play a critical role in ionocytes [13]. NHE can also coordinate with carbonic anhydrase and bicarbonate transporter in *Tribolodon hakonensis* and medaka (*Oryzias latipes*) at acidic environment [14, 15]. Salinity in aquatic environment is an important environmental factor for survival of aquatic animals. The expression level of NHE3 was higher in brackish water than seawater in Atlantic stingray (*Dasyatis Sabina*) and bull shark (*Carcharhinus leucas*) [16, 17]. Besides gills in elasmobranch species, in banded hound shark (*Triakis scyllium*), the expression changes of NHE3 were also detected in the kidney and intestine at different salinity environments [9]. At present, mechanisms of the ammonia nitrogen tolerance have been studied in fish, including inhibiting protein and amino acid catabolism, reducing environmental pH, NH_4_^+^ and NH_3_ continuous excretion, synthesis of non-toxic glutamine, and synthesis of urea, etc. [18–20]. Ammonia is mainly excreted as NH_3_ in zebrafish (*Danio rerio*) and medaka embryos [21, 22]. It is essential for NH_3_ excretion to form NH_4_^+^ via combine H^+^ [21]. Based on the acid-trapping hypothesis of ammonia excretion [23], NH_3_ excretion was promoted in acidic environment which would increase the conversion of NH_3_ to NH_4_^+^. NHE proteins are essential in the process of ammonia excretion [22] and has a combined effect with carbonic anhydrase (CA) and Rhesus-type ammonia transporter (Rh) [2, 24].

The Chinese tapertail anchovy (*Coilia nasus*) is an economically valuable fish widely distributed in China, Japan, and Korea. The sexually mature fish run thousands of kilometers from marine to river [25]. Ion uptake is activated in *C. nasus* during hypotonic environment, and ion excretion and water conservation are promoted in *C. nasus* during hypertonic environment [26]. Moreover, excessive ammonia nitrogen could lead to mass death of *C. nasus* larvae and juvenile during artificial breeding [27]. Based on previous studies, NHEs are involved in osmoregulation [26] and ammonia stress [27] in gills of *C. nasus*. To localize *NHE* genes in the *C. nasus* genome and their functions on salinity challenge and ammonia stress, we identified *NHE* gene family, and detected their expression levels under salinity and ammonia stress. Our findings will provide insight into their effects on ion transport and ammonia tolerance and be beneficial for healthy aquaculture of *C. nasus*.

## Results

### Identification of *NHE* genes

The entire *NHE* gene family, 10 *NHE* genes, were identified in *C. nasus* genome, including *NHE1*, *NHEβ*, *NHE2*, *NHE2-like*, *NHE3*, *NHE5*, *NHE6a*, *NHE6b*, *NHE7*, and *NHE8*. The detail information of *C. nasus NHE* genes were displayed in Table 1.

**Table 1:** The sequence information of *C. nasus NHE* gene family

Gene name	Chromosome location	CDS length (bp)	Exon number	Protein length (aa)	Protein molecular weight (Da)	Isoelectric point (pI)	ID in *C. nasus* genome
*NHE1*	LG19	2220	6	1076	82,508	5.89	augustus-scaffold8-processed-gene-12.25-mRNA-1
*NHEβ*	LG6	1746	8	581	64,374	8.04	maker-scaffold214-augustus-gene-3.54-mRNA-1
*NHE2*	LG12	2610	15	869	98,001	5.27	maker-scaffold134-augustus-gene-2.29-mRNA-1
*NHE2-like*	LG17	2322	11	773	87,191	8.53	augustus-scaffold63-processed-gene-6.3-mRNA-1
*NHE3*	LG6	3231	18	1076	120,098	6.42	maker-scaffold60-augustus-gene-4.23-mRNA-1
*NHE5*	LG18	3549	12	1182	130,950	9.18	maker-scaffold18-augustus-gene-8.24-mRNA-1
*NHE6a*	LG20	2142	14	713	78,416	6.14	maker-scaffold89-augustus-gene-4.19-mRNA-1
*NHE6b*	LG14	1584	6	527	57,652	9.33	maker-scaffold127-augustus-gene-4.25-mRNA-1
*NHE7*	LG1	3297	19	1098	121,677	6.05	maker-scaffold241-augustus-gene-2.55-mRNA-1
*NHE8*	LG21	2373	12	790	87,695	6.95	augustus-scaffold304-processed-gene-2.2-mRNA-1

### Chromosomal distribution of *NHE* genes

**Fig. 1:**
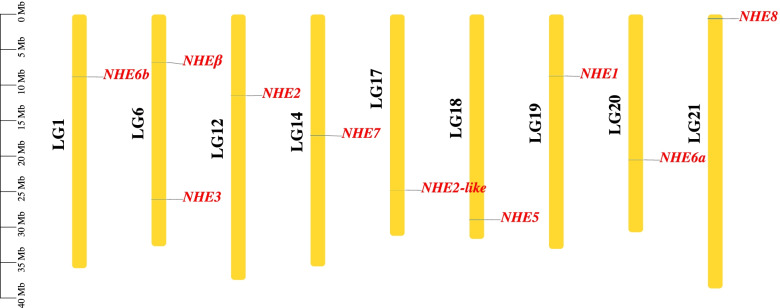
Chromosomal location of *NHE* gene family. The chromosomes were characterized by yellow bars. Chromosome numbers are shown on the left of the chromosomes. *NHE* genes are marked in red on the right of the chromosomes. LG:the name of chromosomes in *C. nasus* genome

*NHE1*, *NHE2*, *NHE2-like*, *NHE5*, *NHE6a*, *NHE6b*, *NHE7*, and *NHE8* were located on chromosome 19 (LG 19), LG 12, LG 17, LG 18, LG 20, LG 1, LG 14, and LG 21, respectively (Fig. 1). *NHEβ* and *NHE3* were located on LG 6 (Fig. 1).

### Phylogenetic analysis

**Fig. 2:**
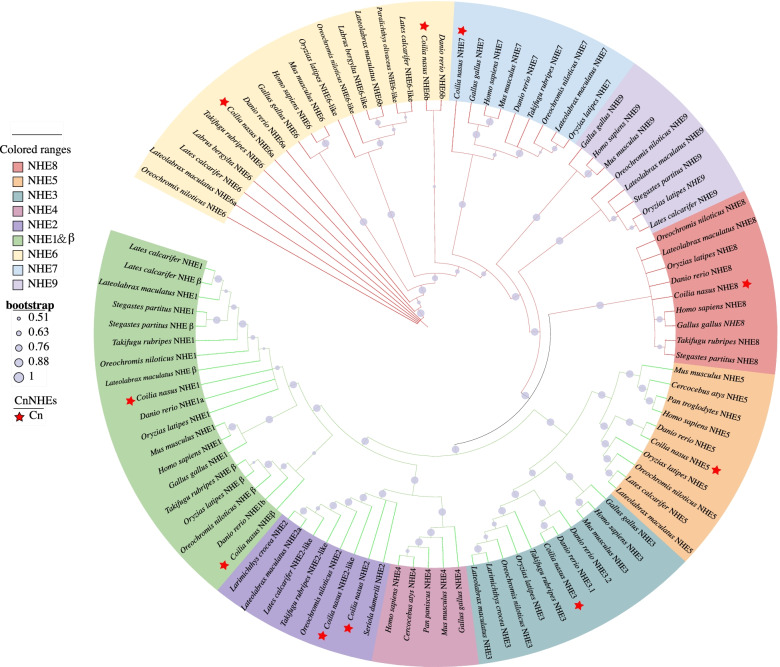
Phylogenetic analyses of NHE proteins from representative vertebrates. The tree was contributed by the neighbor-joining (NJ) method in MEGA X with 1000 bootstrap replications. Bootstrapping values were showed via circles on each branch. *C. nasus* NHEs were highlighted by the red star. The plasmalemmal subgroup (NHEβ, NHE1–5) and the intracellular subgroup (NHE6–9) were differentiated by red and green branches

NHEs can be classified into plasmalemmal subgroup (NHE1–5) and intracellular subgroup (NHE6–9) based on subcellular localization and phylogenic analysis [4]. Our phylogenic analysis showed that *C. nasus* NHEs were categorized into plasmalemmal subgroup (NHE1, NHEβ, NHE2, NHE2-like, NHE3, NHE5) and intracellular subgroup (NHE6a, NHE6b, NHE7, and NHE8) (Fig. 2). Moreover, phylogenic analysis cannot distinguish *C. nasus* NHE1 and NHEβ.

### Structural analysis of the *NHE* genes

**Fig. 3:**
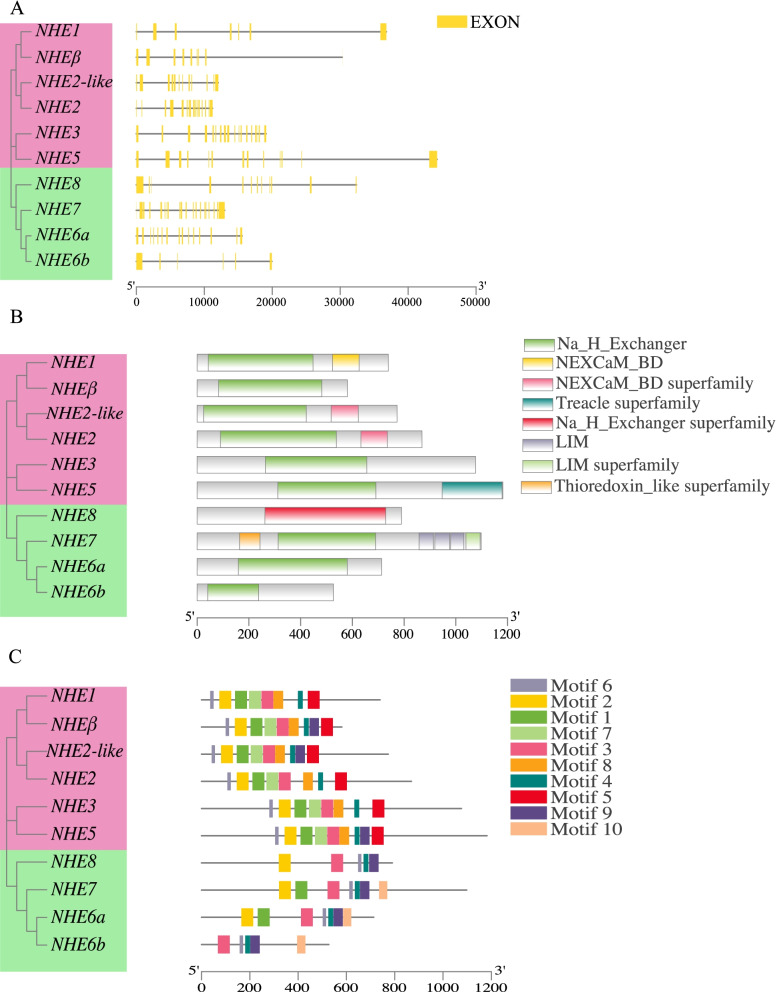
Structural analysis of *C. nasus NHE* genes. **A** gene structure, (**B**) conversed domains and (**C**) motifs. The plasmalemmal subgroup (*NHEβ*, *NHE1–5*), and the intracellular subgroup (*NHE6–8*) were differentiated by pink and green

To further explore the characteristics of *C. nasus NHE* genes, analysis of gene structure, conserved domains and motif was performed. The exon numbers of *NHE1* were the minimum (6 exons), and the exon numbers of *NHE7* owned maximum exons (19 exons) (Table 1, Fig. 3A). All of *C. nasus* NHEs contained the Na_H_Exchanger domain, expect NHE8 which contained the Na_H_Exchanger superfamily domain (Fig. 3B). NHEβ, NHE3, NHE6a, NHE6b, and NHE8 contained only one domain, while others contained two or more domains. Motif analysis showed that motif 3, 4, and 6 existed in all of *C. nasus* NHEs. Noticeably, motif 5 only existed in plasmalemmal subgroup (NHE1, NHEβ, NHE2, NHE2-like, NHE3, NHE5), and motif 10 only existed in intracellular subgroup (NHE6a, NHE6b, and NHE7) (Fig. 3C). The sequences of each motif were shown in Supplementary material: Fig. S1.

### Synteny analysis

**Fig. 4:**
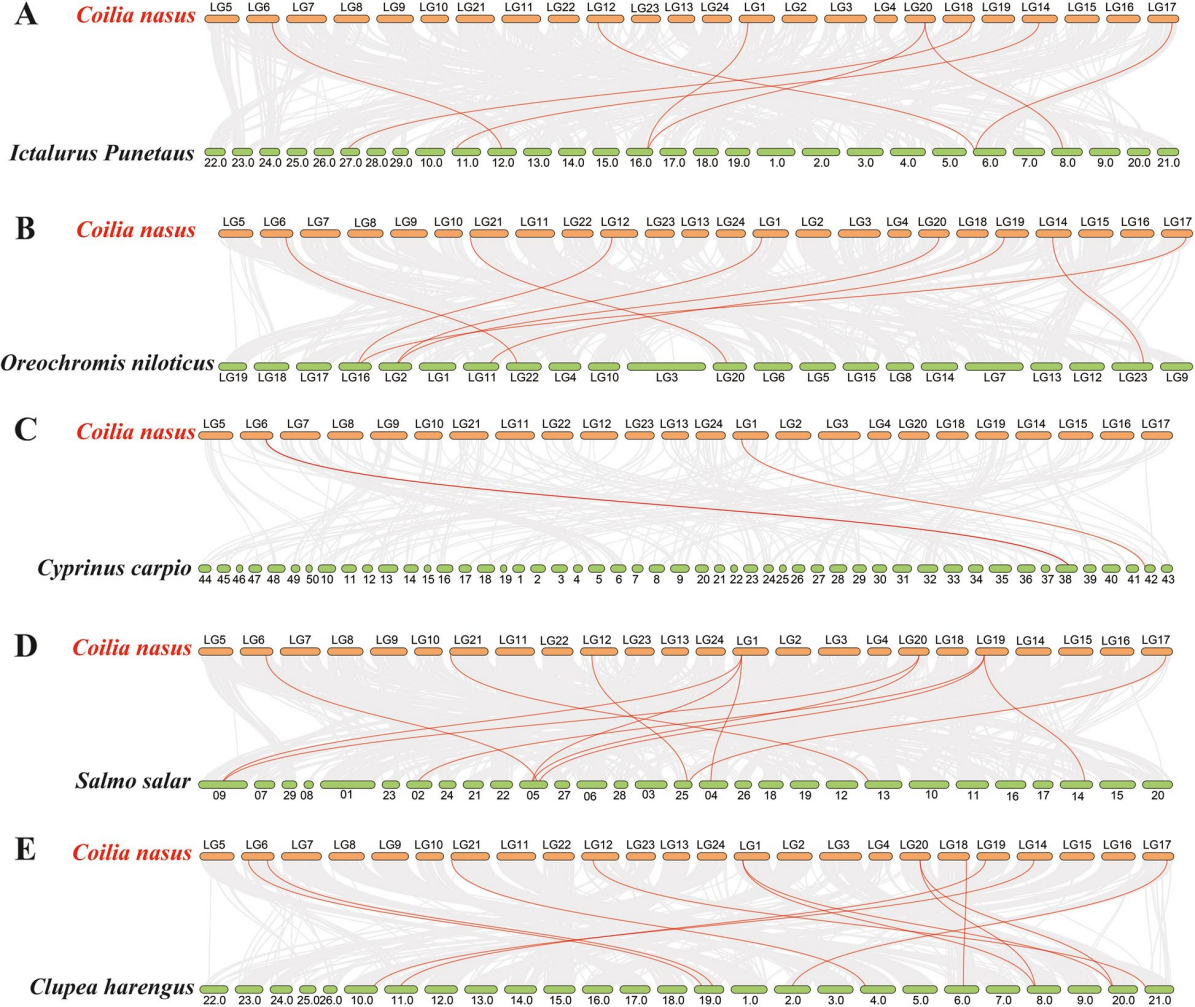
Syntenic analysis of *NHE* genes between *C. nasus* and five other fish species, including *Ictalurus Punetaus* (**A**), *Oreochromis niloticus* (**B**), *Cyprinus carpio* (**C**), *Salmo salar* (**D**), and *Clupea harengus* (**E**). The gray lines indicate the collinear blocks within *C. nasus* and other fish genomes. The red lines indicate the pairs of *NHE* genes

The syntenic analysis was performed to further explore the evolutionary relationship of *NHE* genes between *C. nasus* and other fish species. The number of homolog pairs between *C. nasus NHEs* and other fish species, including channel catfish, Nile tilapia, common carp, Atlantic salmon, and Atlantic herring, were 7, 8, 2, 7, and 10, respectively (Fig. 4A-E).

### Expression profiles *NHEs* of multiple tissues and embryonic development stages

**Fig. 5:**
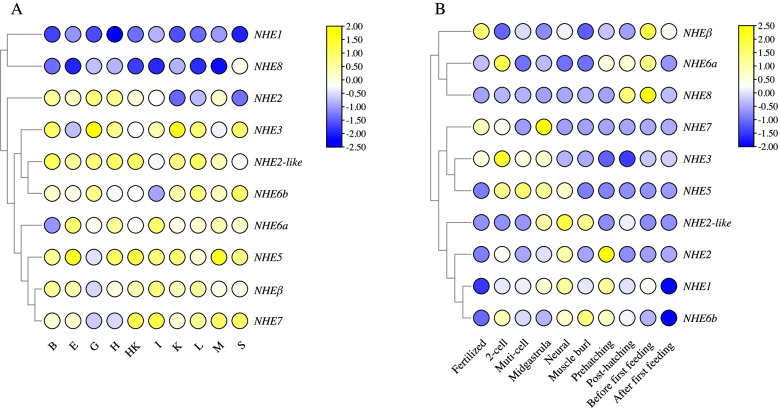
Expression profiles of multiple tissues (**A**) and embryonic development stages (**B**). B: brain, E: eye, G: gill, H: heart, HK: head kidney, K: kidney, I: intestine, L: liver, M: muscle, S: spleen. The expression data were processed by log scale. The branches on the left represent the cluster analysis of different genes from different samples based on their expression patterns. The HK sample of *NHE2* was used as the reference sample in tissues expression, and the 2-cell sample of *NHE1* was used as the reference sample in embryonic development expression

Expression profiles of *NHEs* was detected via qRT-PCR in *C. nasus* brain, eye, gill, heart, head kidney, kidney, intestine, liver, muscle, and spleen (Fig. 5A). *NHE2* displayed higher expression in brain, gill, and heart. *NHE3* displayed higher expression in brain, gill, and kidney. High expression level of *NHE2-like* was displayed in brain, eye, gill, heart, head kidney, kidney, liver, and muscle. High expression level of *NHE5* was displayed in eye, heart, head kidney, kidney, intestine, and muscle. High expression level of *NHE6a* was displayed in eye, heart, and heart. *NHE6b* displayed higher expression in gill, liver, and spleen. *NHE7* displayed higher expression in head kidney, intestine, liver, muscle, and spleen. *NHEβ* displayed higher expression in brain, eye, intestine, and liver. The expression of *NHE1* and *NHE8* were almost undetectable in all tissues used in our study.

Expression profiles of *NHEs* was detected via qRT-PCR at fertilized stage, 2-cell stage, multi-cell stage, midgastrula stage, neural stage, muscle burl stage, prehatching, post-hatching, before first feeding, and after first feeding (Fig. 5B). *NHEβ* displayed higher expression at fertilized stage and before first feeding. *NHE6a* displayed higher expression at 2-cell stage and before first feeding. *NHE8* displayed highest expression at post-hatching stage and before first feeding. High expression level of *NHE7* was displayed in fertilized stage and midgastrula stage. High expression level of *NHE3* was displayed in 2-cell stage. High expression level of *NHE5* was displayed in 2-cell stage, multi-cell stage, midgastrula stage, and neural stage. High expression level of *NHE2-like* was displayed in midgastrula stage, neural stage, and muscle burl stage. *NHE2* displayed highest expression at neural stage, and prehatching stage. *NHE1* displayed higher expression at midgastrula stage, neural stage, and prehatching stage. *NHE6b* displayed higher expression at 2-cell stage, neural stage, muscle burl stage, prehatching.

### Expression of *NHEs* in response to salinity challenge and ammonia stress

**Fig. 6:**
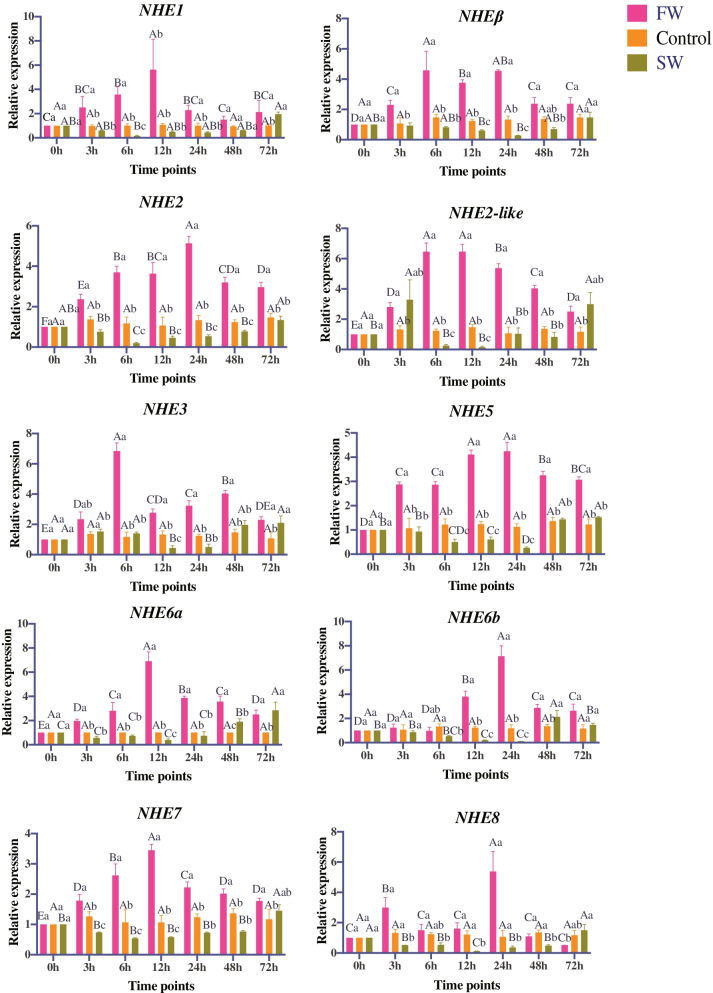
Expression profiles of *NHE* genes in *C. nasus* gills in response to hypotonic (FW vs Control) and hypertonic (SW vs Control) stress at multiple time points. Different capital letters indicate significant difference among different time points in the same groups at *P* < 0.05. Different lower-case letters indicate significant difference between different groups at the same time point at *P* < 0.05. FW: salinity ~ 1 ppt, Control: salinity 10 ppt, SW: salinity 30 ppt. The 0 h sample of Control group was used as the reference sample

To explore *C. nasus NHEs* in response to hypotonic stress and hypertonic stress, their expression profiles were detected under hypotonic stress (F vs C) and hypertonic stress (S vs C) in the gill via qRT-PCR (Fig. 6). Overall, expression profiles of all *NHEs* were significantly upregulated at first, and then significantly downregulated under hypotonic stress. The converse trend was displayed in hypertonic stress. *NHEs* were significantly downregulated at first, and then significantly upregulated. During hypotonic stress, *NHE1*, *NHE2-like*, and *NHE3* showed highest expression at 6 h. *NHE6a* and *NHE7* showed highest expression at 12 h*. NHEβ*, *NHE2*, *NHE5*, *NHE6b*, and *NHE8* showed highest expression at 24 h. During hypertonic stress, *NHE1*, *NHE2*, *NHE7* showed lowest expression at 6 h. *NHE2-like*, *NHE3*, *NHE6a*, and *NHE8* showed lowest expression at 12 h. *NHEβ*, *NHE5*, and *NHE6b* showed lowest expression at 24 h.

**Fig. 7:**
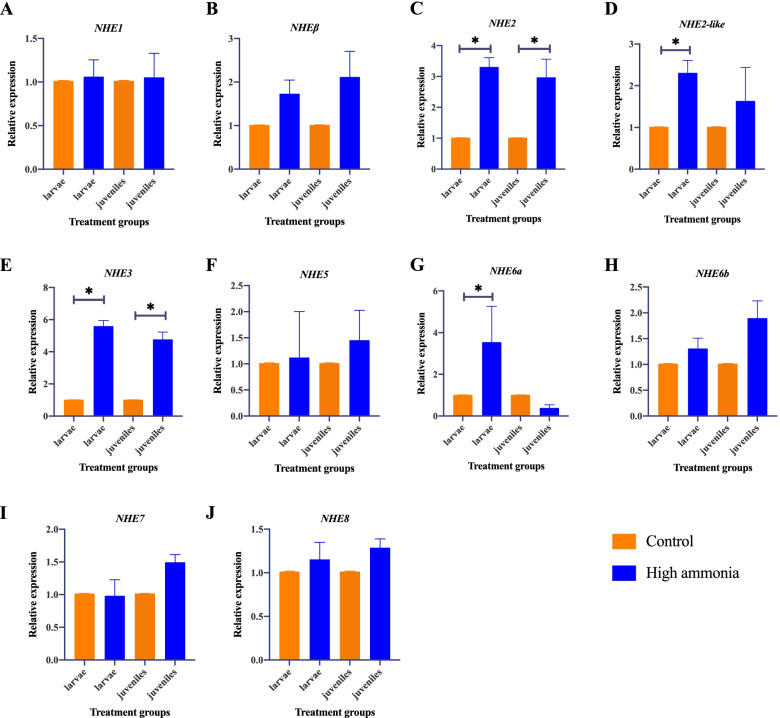
Expression patterns of *NHE* genes in *C. nasus* larvae and juvenile gills responding to ammonia stress. Significant differences were noted by asterisk (*P* < 0.05). The samples of larvae and juveniles in control group were used as reference sample, respectively

To explore the potential functions of *NHEs* of *C. nasus* larvae and juveniles in response to ammonia stress, their expression patterns were detected in the gill via qRT-PCR (Fig. 7). In *C. nasus* larvae, the expression of *NHE2*, *NHE2-like*, *NHE3*, and *NHE6a* were significantly enhanced under ammonia stress, while other *NHEs* displayed no significant difference. In juveniles, the expression of *NHE2* and *NHE3* were significantly enhanced under ammonia stress, while other *NHEs* displayed no significant difference.

## Discussion

Ten *NHE* genes were identified in *C. nasus.* Based on analysis of other vertebrates *NHE* gene family, *NHEβ* was only identified in teleost species, and *NHE4* is generally missing in teleost fishes [28–30], which was generally identified in mammals [2]. In the present study, *NHEβ* was identified, and *NHE4* missed in *C. nasus NHE* gene family. Furthermore, *C. nasus NHE9* was not identified*.* As the oldest NHE gene, *NHE9* lost in some fish [31]. Moreover, the syntenic analysis showed that the number of homolog pairs between *C. nasus NHEs* and other fish species, including channel catfish, Nile tilapia, Atlantic salmon, and Atlantic herring, were 7, 8, 7, and 10, respectively, which indicated that the genetic relationship between *C. nasus* and these fish species was close. The genetic relationship between *C. nasus* and common carp is closer than channel catfish and Nile tilapia based on traditional fish taxonomy, while homolog pairs of *NHEs* between *C. nasus* and common carp is only 2. Common carp is stenohaline fish, while other fish species are euryhaline fishes. These results suggested that most of *NHEs* between stenohaline fish and euryhaline fish are not conserved, or most of *NHEs* has been lost in stenohaline fish genome.

Based on tissue-specific expression patterns, *NHE2* and *NHE3* displayed higher expression levels in *C. nasus* gills. *NHE2* and *NHE3* are the primary isoforms expressed in gills of multiple fishes, including zebrafish [32], Pacific dogfish (*Squalus suckleyi*) [33], rainbow trout (*Oncorhynchus mykiss*) [34, 35], and *Fundulus heteroclitus* [36], which was similar to our results. In winter flounder (*Pseudopleuronectes americanus*) and zebrafish, *NHE1* displayed high expression levels in red blood cells [32, 37]. Therefore, the expression level of *NHE1* was almost undetectable in all tissues used in our study. However, few research focused on tissue distribution other *NHEs*. *NHE5* and *NHE6* in the brain, and *NHE7* in the testis were three *NHEs* with higher expression levels in zebrafish [32]. *NHE6b* and *NHE9* showed higher expression levels in the spleen, and *NHE8* higher expression levels in the liver of European sea bass (*Lateolabrax maculatus*) [2]. These results were different from our results, which implied that the tissue distributions of *NHEs* were different from different fish species considering the habitats and lifestyle. *C. nasus NHEs* displayed different expression patterns during multiple embryonic development stages. Yolk proteins and amino acids are the main energy source in most teleost fish during embryonic development [38–40]. Their metabolism can continuously produce a waste product, ammonia. NHEs involved in ammonia excretion and Na^+^ uptake [41, 42]. NHE3 can induce Rhesus glycoprotein (Rh) proteins to involve in ammonia excretion during embryonic development stage in *Coryphaena hippurus* [43]. Similar results have been reported in medaka, zebrafish, and rainbow trout [42, 44]. Moreover, as a consequence, embryos of oviparous organisms are exposed to high respiratory CO_2_ within the egg capsule due to their increasing metabolic rate and the egg capsule wall acting as a diffusion barrier [45, 46]. High respiratory CO_2_ can decrease pH in the embryo and larvae. In acid-secreting ionocytes, NHEs are believed to be specialized in the secretion of acid equivalents [47]. It is proposed that ammonia transporters from the Rh family in combination with NHE3, expressed in HR cells are key players in mediating the active secretion of ammonia and protons in seawater teleost [22, 48]. A previous study demonstrated that NHE3 expressing epidermal ionocytes of cephalopod embryos are also involved in active secretion of acid equivalents [49]. Together, different expression patterns of *C. nasus NHEs* during multiple embryonic development stages seem to involve in ammonia excretion, Na^+^ uptake, and maintaining cellular pH homeostasis, or other physiological processes.

Gill is an indispensable tissue in fish, playing critical roles in osmoregulation and ammonia excretion. NHE is a two-way ion exchange carrier protein, which can transport Na^+^ into cells and exchange out H^+^ (or NH4^+^) [50, 51]. The expression of *NHE3* was increased in the apical membrane of mitochondria-rich cells of *Dasyatis sabina* under low-salinity stress, thereby promoting the absorption of Na^+^ [16]. The mRNA expression level of *NHE3* in Mozambique tilapia (*Oreochromis mossambicus*) gills in freshwater environment is higher than that in seawater environment [52]. Similar results have been reported in other fish, including *D. sabina* [16], zebrafish [32], banded hound shark [9], and Pacific dogfish [33]. In the present study, *NHE2*, *NHE2-like* and *NHE3* with higher expression in the gills were displayed in *C. nasus* under hypotonic stress, which was consistent with other fish species. However, they were downregulated in the gills of *C. nasus* under hypertonic stress. This finding was also occurred in European sea bass [2]. Besides *NHE2* and *NHE3*, expression levels of other *NHE* genes were also detected, and they showed higher expression levels in the gills of *C. nasus* under hypotonic stress and lower expression levels in hypertonic stress, which was similar to European sea bass [2]. These results indicated that *C. nasus NHEs* appear to have differing functions in hypotonic and hypertonic regulation via exchanging extracellular Na^+^ for intracellular H^+^.

Besides osmoregulation, NHEs are also essential for ammonia excretion. In the present study, the expression levels of *NHE2*, *NHE2-like*, *NHE3*, and *NHE6a* were significantly upregulated in *C. nasus* larvae gills under ammonia stress for 24 h. And the mRNA expression of *NHE2* and *NHE3* were significantly upregulated in *C. nasus* juvenile gills. Similarly, the mRNA expression of *NHE2* and *NHE3* were upregulated in *Boleophthalmus pectinirostris* under ammonia stress [41]. The mRNA expression level of *NHE* was upregulated more significantly in *B. pectinirostris* than *Periophthalmus magnuspinnatus* subjected to treatment with high environmental ammonia for 72 h [53]. Based on transport physiology, it is generally believed that NH_3_ and CO_2_ move across biological membranes to a much higher degree via membrane channels than simple, passive diffusion [54]. Ammonia excretion in fishes occurs via a “Na^+^/NH_4_^+^-exchange metabolon” which involves NHEs [22, 41, 55]. NH_3_ can diffuse from cells into water via Rh glycoproteins. As soon as it enters the water, NH_3_ combines with H^+^ which is pumped from the gill cell by H^+^-ATPase and/or by one or more NHE proteins, to form NH4^+^ [41]. There is indirect coupling of NH_4_^+^ efflux to Na^+^ uptake by either of these H^+^ efflux mechanisms. These results implied that significant up-regulation of *NHE* in *C. nasus* larvae and juveniles promoted NH_3_ to form NH_4_^+^ via pumping H^+^ into water to reduce ammonia toxicity. Moreover, the expression levels of Rhcg1, H^+^-ATPase, NHE, Na^+^/Ka^+^-ATPase (NKA), and Na^+^/Ka^+^/Cl^−^ cotransporter (NKCC) were upregulated in *Takifugu rubripes* exposed to high external ammonia, and they showed combined effects on ammonia excretion [48]. The combined effects between NHEs and other proteins are required to further study in *C. nasus* under ammonia stress.

## Conclusions

In the present study, 10 *NHE* genes were systematically identified from *C. nasus* genome. Phylogenetic analysis showed that *NHE4* and *NHE9* were lost in *C. nasus* genome. Syntenic analysis showed significant difference between stenohaline fish and euryhaline fishes. Different expression patterns of *C. nasus NHE* genes were displayed in multiple tissues. Different expression patterns of *C. nasus NHE* genes during multiple embryonic development stages were related to ammonia excretion. During hypotonic stress, *C. nasus NHE* genes were significantly upregulated. During hypertonic stress, they were significantly downregulated. During ammonia stress, *NHE2* and *NHE3* were significantly upregulated in *C. nasus* larvae and juveniles. These studies will provide insights into molecular mechanism of osmoregulation and ammonia tolerance in teleost.

## Methods and materials

### Identification of NHE gene family in *C. nasus*

To identify *NHE* gene family in *C. nasus*, the whole genome databases (GenBank GCA_007927625.1) were searched using BLAST GUI Wrapper on TBtools (v1.0692) according to amino sequences of human (*Homo sapiens*), zebrafish, and Atlantic herring (*Clupea harengus*) downloaded from Ensembl (http://www.ensembl.org) and NCBI (http://www.ncbi.nlm.nih.gov/) databases (cutoff value <1e-5). After removing repeated sequences, the unique sequences were validated via BLASTN against NCBI non-redundant protein database. Moreover, the Na_H_Exchanger domain (PF00999.21) was downloaded on Pfam database (http://pfam.xfam.org/). The Simple HMM search on TBtools (v1.0692) was used for seeking *C. nasus* NHE proteins with E-value < 0.01. The generated NHE proteins were verified on the Pfam databases. After removing the repeated sequences sought from the two methods above, the unique sequences were retained for further analysis.

The chromosomal location was performed based on their locations on *C. nasus* genome via Gene Location Visualize (Advanced) on the TBtools (v1.0692). the molecular weight and pI of NHE family were detected on ExPASy-Compute pI/Mw tool (https://web.expasy.org/compute_pi/).

### Phylogenetic analysis of NHEs

The phylogenetic analysis of NHE sequences between *C. nasus* and several vertebrates were performed by MEGA X software. The accession numbers used for phylogenetic analysis were listed on Supplementary Table S1. The phylogenetic tree beautification was performed beautified on Interactive Tree of Life (iTOL, http://itol.embl.de/).

### Structure analysis of *NHEs*

The gene structure of *NHE* genes were analyzed based on *C. nasus* genome database (gff3 file). Conserved domains of NHEs were analyzed via Conserved Domain Search Service (https://www.ncbi.nlm.nih.gov/Structure/cdd/wrpsb.cgi). Motifs of NHEs were analyzed via Multiple Em for Motif Elicitation (MEME) (https://meme-suite.org/meme/tools/meme). Gene Structure View (Advanced) on TBtools was used for the visualization of gene structure, conversed domains, and motifs.

### Synteny analysis of *NHEs*

One Step MCScanX on TBtools was used to examine gene duplication [56, 57]. Homology of *NHE* genes was analyzed between *C. nasus* and other five fishes, including channel catfish (*Ictalurus Punetaus*), Nile tilapia (*Oreochromis niloticus*), common carp (*Cyprinus carpio*), Atlantic salmon (*Salmo salar*), and Atlantic herring. Dual Systeny Plot for MCScanX on TBtools was used for the visualization of synteny analysis.

### Salinity challenge, ammonia stress, and samples collection

Healthy *C. nasus* (5.54 ± 0.63 cm, 2.25 ± 0.83 g) used in the present study were from Jiangzhiyuan Fishery Technology Co., Ltd. (Yangzhong, China). Before treatments, the salinity of aquatic environment was kept at 10 ppt for two weeks until salinity challenge experiment begins using NaCl. After acclimation, 180 *C. nasus* were randomly allocated into three groups (in triplicates): control group (C, salinity 10 ppt), hypotonicity group (F, salinity ~ 1 ppt), and hypertonicity group (S, salinity 30 ppt). After exposure for 0 hour (h), 3 h, 6 h, 12 h, 24 h, 24 h, 48 h, and 72 h, the treated fish were anesthetized via 70 mg/L buffered tricaine methanesulfonate (MS-222) (Greenhengxing, Beijing, China), and their gills were immediately collected and then stored at − 80 °C for further molecular assays.

Healthy *C. nasus* larvae (2.33 ± 0.25 cm, 1.11 ± 0.21 g) and juveniles (5.12 ± 0.45 cm, 2.35 ± 0.47 g) were reared for 10 days (27 ± 1.5 °C, pH 8.0 ± 0.4, salinity < 1.7, ammonia nitrogen < 0.005 mg/L). After acclimation, larvae and juveniles were randomly allocated into control group (ammonia nitrogen < 0.005 mg/L) and ammonia stress group (concentration of ammonia ~ 280 umole/L) (in triplicates), respectively. Stock solution (1.0 mol/L) of high purity NH_4_Cl was used as the source of the total ammonia. After exposed for 24 hours (h) (28 ± 2.1 °C, pH 7.5 ± 0.3, salinity < 2.3), gills were immediately collected and then stored at − 80 °C for further molecular assays.

For analysis of tissue distribution, three *C. nasus* (24.7 ± 1.68 cm, 11.9 ± 0.62 g) were anesthetized via 70 mg/L buffered MS-222, and then eye, gill, brain, liver, spleen, intestines, heart, head-kidney, kidney, and muscle were immediately collected and then stored at − 80 °C for further molecular assays. Tissue samples from all 3 fish were pooled together to make one sample. For ontogenetic expression profiles, embryos and larvae during multiple developmental stages were collected following natural spawning of the brood stock. Every developmental stage was examined by microscope observation. 30 embryos at fertilized egg (0 hour post fertilization (hpf)), 2-cell (1 hpf), muti-cell (3 hpf), midgastrula (4 hpf), neural (11 hpf), muscle burl (21 hpf), prehatching (28 hpf), and post-hatching (30 hpf); 20 larvae before first feeding (96 hpf) and after first feeding (144 hpf) were immediately pooled and frozen in liquid nitrogen.

### Quantification of the *NHEs* expression by quantitative real time-PCR (qRT-PCR)

cDNA was synthesized using the PrimeScript™ RT Reagent Kit (TaKaRa, Tokyo, Japan). Primer Premier 5 software was used to design the primers used for qRT-PCR (Supplementary material: Table S2). The reactions were carried out on the Bio-Rad CFX96 real-time PCR system (Bio-Rad, Hercules, CA, USA). The reaction system (20.0 μL) included 10.0 μL of iTaq™ Universal SYBR® Green Supermix (Bio-Rad), 2.0 μL of cDNA, 1.0 μL of each primer (10 μmol/L), and 6.0 of PCR-grade DEPC water. Reactions were performed in triplicate per sample, and cycling parameter was set as following: 94 °C for 2 min, followed by 40 cycles of 15 s at 94 °C,30 s at 60 °C, and 45 s at 72 °C. The geometric means of *β-actin*, *18SrRNA*, and *GAPDH*, housekeeper genes were used to normalize expression levels of *NHE* genes [58]. All samples were detected in triplicate and the relative expression levels of *NHE* genes were calculated using the 2^-ΔΔCT^ method [59].

### Statistical analysis

The data of *C. nasus NHEs* expression during salinity and ammonia stress were analyzed by two-way ANOVA. Kolmogorov-Smirnov and Shapiro-Wilk methods were used to test the normal distribution. Interactive effect was tested using conversed non-normally distributed data. When *P* < 0.05, two-way ANOVA was performed. Data analysis was performed on SPSS 20.0. All data were displayed as mean ± SE. *P* < 0.05 indicates a significant difference. Histograms were drawn via GraphPad 8.0.

## Supplementary Information

**Additional file 1.**

## Data Availability

The datasets generated and analysed during the current study are available in the NCBI database (https://www.ncbi.nlm.nih.gov/assembly/GCA_007927625.1/).
