# Supplementary material for: Genome-wide identification of the NHE gene family in Coilia nasus and its response to salinity challenge and ammonia stress
Source: BMC Genomics. 2022 Jul 20;23:526. doi: 10.1186/s12864-022-08761-9 (PMC9297642; doi:10.1186/s12864-022-08761-9)
Supplement: Supplementary file 1 — Additional file 1. [file 12864_2022_8761_MOESM1_ESM.docx]

Table S1. Gene names and accessions numbers of NHEs used in the present study

| Gene name | Accession number | Scientific Name |
| --- | --- | --- |
| NHE1 | NP_001038108.1 | *Gallus gallus* |
| NHE1 | XP_003965701.1 | *Takifugu rubripes* |
| NHE1 | XP_020565979.1 | *Oryzias latipes* |
| NHE1 | NP_058677.1 | *Mus musculus* |
| NHE1 | XP_005455375.1 | *Oreochromis niloticus* |
| NHE1 | NP_003038.2 | *Homo sapiens* |
| NHE1 | AXV43377.1 | *Lateolabrax maculatus* |
| NHE1a | XP_005173599.2 | *Danio rerio* |
| NHE1 | XP_008293192.1 | *Stegastes partitus* |
| NHE1 | XP_018555519.1 | *Lates calcarifer* |
| NHE1b | NP_001106952.1 | *Danio rerio* |
| NHEbeta | XP_008293191.1 | *Stegastes partitus* |
| NHEbeta | XP_018555518.1 | *Lates calcarifer* |
| NHEbeta | XP_003968869.1 | *Takifugu rubripes* |
| NHEbeta | XP_023815746.1 | *Oryzias latipes* |
| NHEbeta | XP_003447330.1 | *Oreochromis niloticus* |
| NHEbeta | AXV43386.1 | *Lateolabrax maculatus* |
| NHE2-like | XP_011606645.1 | *Takifugu rubripes* |
| NHE2 | XP_005457888.1 | *Oreochromis niloticus* |
| NHE2 | XP_010752680.3 | *Larimichthys crocea* |
| NHE2a | AXV43378.1 | *Lateolabrax maculatus* |
| NHE2-like | XP_018557812.1 | *Lates calcarifer* |
| NHE2-like | XP_022616058.1 | *Seriola dumerili* |
| NHE3 | XP_004935132.1 | *Gallus gallus* |
| NHE3 | XP_011607634.1 | *Takifugu rubripes* |
| NHE3 | XP_023815787.1 | *Oryzias latipes* |
| NHE3 | NP_001074529.1 | *Mus musculus* |
| NHE3 | XP_025758291.1 | *Oreochromis niloticus* |
| NHE3 | AXV43379.1 | *Lateolabrax maculatus* |
| NHE3 | NP_004165.2 | *Homo sapiens* |
| NHE3.1 | NP_001106944.1 | *Danio rerio* |
| NHE3.2 | XP_009292331.1 | *Danio rerio* |
| NHE3 | XP_010740588.2 | *Larimichthys crocea* |
| NHE4 | NP_001244184.1 | *Gallus gallus* |
| NHE4 | NP_796058.1 | *Mus musculus* |
| NHE4 | NP_001011552.2 | *Homo sapiens* |
| NHE4 | XP_003804498.1 | *Pan paniscus* |
| NHE4 | XP_011910887.1 | *Cercocebus atys* |
| NHE5 | XP_023809551.1 | *Oryzias latipes* |
| NHE5 | NP_001074801.1 | *Mus musculus* |
| NHE5 | XP_019216873.2 | *Oreochromis niloticus* |
| NHE5 | AXV43380.1 | *Lateolabrax maculatus* |
| NHE5 | NP_004585.1 | *Homo sapiens* |
| NHE5 | NP_001106943.1 | *Danio rerio* |
| NHE5 | XP_016785517.1 | *Pan troglodytes* |
| NHE5 | XP_011918539.1 | *Cercocebus atys* |
| NHE5 | XP_018548328.1 | *Lates calcarifer* |
| NHE6 | XP_015133960.3 | *Gallus gallus* |
| NHE6 | XP_011608738.2 | *Takifugu rubripes* |
| NHE6 | XP_011478246.1 | *Oryzias latipes* |
| NHE6 | NP_001345790.1 | *Mus musculus* |
| NHE6 | XP_003445201.1 | *Oreochromis niloticus* |
| NHE6a | MH_687075 | *Lateolabrax maculatus* |
| NHE6 | NP_001036002.1 | *Homo sapiens* |
| NHE6a | NP_001091726.2 | *Danio rerio* |
| NHE6 | XP_018515762.1 | *Lates calcarifer* |
| NHE6 | XP_020491394.1 | *Labrus bergylta* |
| NHE6-like | XP_003447049.2 | *Oreochromis niloticus* |
| NHE6b | AXV43381.1 | *Lateolabrax maculatus* |
| NHE6b | NP_001106947.1 | *Danio rerio* |
| NHE6-like | XP_018546580.1 | *Lates calcarifer* |
| NHE6-like | XP_020506772.1 | *Labrus bergylta* |
| NHE6-like | XP_019952907.1 | *Paralichthys olivaceus* |
| NHE7 | XP_015133243.2 | *Gallus gallus* |
| NHE7 | XP_011612806.1 | *Takifugu rubripes* |
| NHE7 | XP_020558315.1 | *Oryzias latipes* |
| NHE7 | NP_796327.1 | *Mus musculus* |
| NHE7 | XP_005478830.3 | *Oreochromis niloticus* |
| NHE7 | AXV43382.1 | *Lateolabrax maculatus* |
| NHE7 | NP_115980. | *Homo sapiens* |
| NHE7 | NP_001025248.2 | *Danio rerio* |
| NHE8 | NP_001034364.1 | *Gallus gallus* |
| NHE8 | XP_003963383.1 | *Takifugu rubripes* |
| NHE8 | XP_004070550.2 | *Oryzias latipes* |
| NHE8 | XP_019205377.1 | *Oreochromis niloticus* |
| NHE8 | AXV43383.1 | *Lateolabrax maculatus* |
| NHE8 | NP_056081.1 | *Homo sapiens* |
| NHE8 | NP_001008586.1 | *Danio rerio* |
| NHE8 | XP_008298618.1 | *Stegastes partitus* |
| NHE9 | NP_001026476.1 | *Gallus gallus* |
| NHE9 | XP_023805856.1 | *Oryzias latipes* |
| NHE9 | NP_808577.3 | *Mus musculus* |
| NHE9 | XP_005448955.1 | *Oreochromis niloticus* |
| NHE9 | AXV43384.1 | *Lateolabrax maculatus* |
| NHE9 | NP_775924.1 | *Homo sapiens* |
| NHE9 | XP_018529277.1 | *Lates calcarifer* |
| NHE9 | XP_008290941.1 | *Stegastes partitus* |

Table S2. Primers used in present study for qRT-PCR.

| Gene | Primer | Sequence (5’- 3’) | Product length (bp) |
| --- | --- | --- | --- |
| *β-actin* | Forward | AACGGATCCGGTATGTGCAAAGC | 110 |
|  | Reverse | GGGTCAGGATACCTCTCTTGCTCTG | |
| *18SrRNA* | Forward | TGATTGGGACTGGGGATTGAA | 114 |
|  | Reverse | TAGCGACGGGCGGTGTGT | |
| *GAPDH* | Forward | AGCTTGCCACCCTCTTGCT | 121 |
|  | Reverse | AGCCATCAACGACCCCTTC | |
| *NHE1* | Forward | TTCCTGGTGGTGGCAATAGG | 195 |
|  | Reverse | CACCACTCCACAGGCAATCA | |
| *NHEβ* | Forward | CTCTTCACGGAGAACGTGGG | 150 |
|  | Reverse | AAAGATCAGGCACGACAGCA | |
| *NHE2* | Forward | GCTTTCACGTCACGGTTCAC | 135 |
|  | Reverse | TAGGGTGCACACCACAATGG | |
| *NHE2-like* | Forward | GCCTGACGATCCCATAACCA | 154 |
|  | Reverse | GGATTCGGGCACCCAGATAG | |
| *NHE3* | Forward | CATGCCACACCGAACATCAC | 116 |
|  | Reverse | GCCTCTAACTTGCAGAGCCA | |
| *NHE5* | Forward | CGGGATTTCTGCGGTGGATA | 147 |
|  | Reverse | TTGTCCAGGGGAACCAATCG | |
| *NHE6a* | Forward | CGGGGCTTTCCTGGGTATTT | 157 |
|  | Reverse | GATGGACAGGGCAAAGGTCA | |
| *NHE6b* | Forward | CAGACGCGGTTATGGGAACT | 118 |
|  | Reverse | GCACACGTCTCCTCATCACA | |
| *NHE7* | Forward | TCATCGTCGGGGCTTTTCTT | 200 |
|  | Reverse | GAACATCATCTGTCGGGCGT | |
| *NHE8* | Forward | AGCATTCCACACAAGTCGGT | 104 |
|  | Reverse | CATCTATGGTGGCTGGTGGG | |


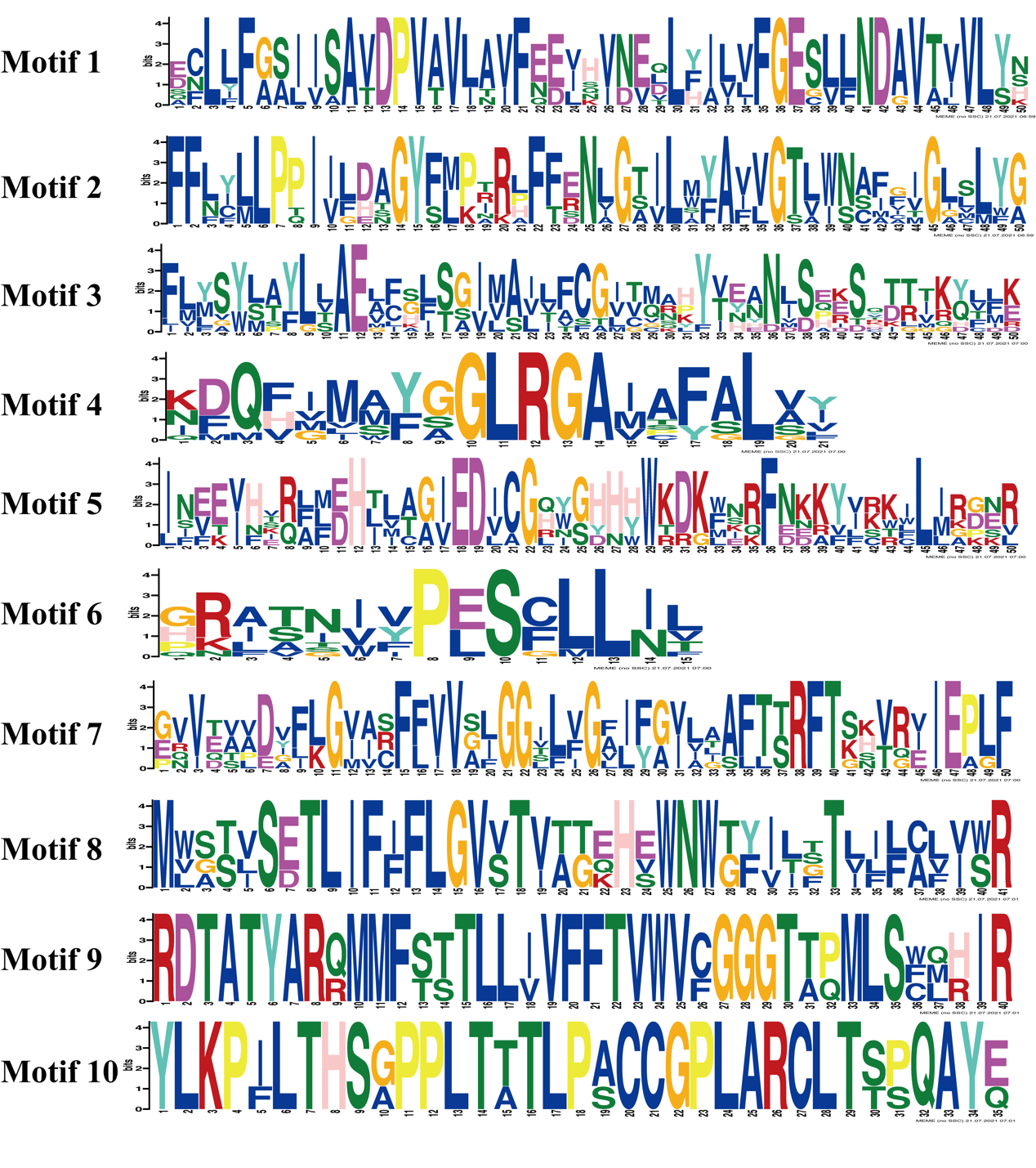


Fig S1. Motif sequences identified in NHE proteins of *C. nasus*. The height of different amino acids represents repeatability. The scale bar at the bottom indicates the length of the motif protein sequence.
